# Commentary on “Failer et al. (2022) Developmental endothelial locus-1 protects from hypertension-induced cardiovascular remodeling via immunomodulation” J Clin Invest 2022 (https://doi.org/10.1172/JCI126155)

**DOI:** 10.1007/s00424-022-02723-6

**Published:** 2022-06-28

**Authors:** Markus Sperandio

**Affiliations:** Institute of Cardiovascular Physiology and Pathophysiology, Walter Brendel Center for Experimental Medicine, Biomedical Center (BMC), LMU München, Großhaderner Str. 9, 82152 Planegg-Martinsried, Germany

Hypertension is a very frequent chronic disease associated with significant cardiovascular morbidity and mortality. Pathological findings in patients with chronic hypertension include aortic fibrosis and loss of elastic fibers leading to arterial stiffening which further increases systolic blood pressure. Chronically elevated blood pressure eventually also affects the heart with the development of left ventricular hypertrophy and fibrosis [1]. An essential component in the pathophysiology of hypertension is inflammation which can be triggered by various mechanisms including chronically elevated angiotensin II levels through activation of the renin–angiotensin–aldosterone system [1]. Accordingly, recruitment of neutrophils and monocytes as well as T-cells into the arterial wall has been well-documented in patients with hypertension as well as in various animal models, including angiotensin II–induced hypertension [2]. In this context, IL-17 turned out to be a critical pro-inflammatory mediator in hypertension-induced inflammation [3, 4]. Recently, developmental endothelial locus-1 (DEL-1) encoded by *EDIL3* has been described to be a negative regulator of IL-17-dependent pathologies [5]. In addition, DEL-1 significantly impacts on neutrophil homeostasis, recruitment, and clearance and promotes FOXP3^+^ regulatory CD4^+^ T-cell differentiation during inflammation [6]. These anti-inflammatory effects are mostly exerted via the inhibition of β_2_ integrins and modulation of α_v_β_3_ integrin function [5].

Hypothesizing that therapeutic use of DEL-1 might be an interesting and effective way to attenuate hypertension-induced cardiovascular remodeling, Failer and colleagues performed an excellent study to address a potential beneficial effect of therapeutic DEL-1 in hypertension-induced cardiovascular disease [7]. The authors used two models of hypertension (angiotensin-II–induced and deoxycorticosterone acetate (DOCA)-salt–induced hypertension) and applied various ways of DEL-1 treatment including the use of endothelial cell–specific DEL-1 overexpressing mice as well as injection of recombinant DEL-1. While in both hypertension models the increase in blood pressure during the early phase of induced hypertension was independent of DEL-1 treatment, the later increase in systolic blood pressure was significantly reduced in the presence of DEL-1. This was accompanied by reduced vascular and cardiac remodeling exemplified by less pronounced aortic stiffening and attenuated LV hypertrophy. These findings strongly suggest that the inflammatory response that takes place during hypertension contributes to disease progression (with α_v_β_3_ integrin and activated MMP2 playing major roles), opening new avenues in treating patients with chronic hypertension. This might be true for both the early and later phases of the disease when vascular and cardiac remodeling has already progressed to substantial changes in vascular and left ventricular tissue architecture. Although the microcirculation was not the focus of the study by Failer et al. [7], arterial vessels in the microcirculation exposed to hypertension might also benefit from DEL-1 treatment in terms of arterial wall hypertrophy, vascular tone regulation, and inflammation-induced alterations of the vascular wall. Antiadhesive properties by DEL-1 had already been reported some time ago in a microcirculatory model (dorsal skinfold chamber), where the loss of DEL-1 facilitates LFA-1 binding to ICAM-1 leading to an increase in leukocyte adhesion to the inflamed endothelium [8]. Additional experimental studies clarifying the role of DEL-1 in the microvasculature exposed to hypertension may further support a protective role of DEL-1 in the microcirculation of patients with chronic hypertension and promote the use of DEL-1 treatment in those patients.

Taken together, Failer and colleagues present convincing evidence that DEL-1-dependent modulation of the inflammatory response during chronic hypertension might offer an interesting new treatment strategy to complement conventional antihypertensive therapy [7]. Applied as antihypertensive and anti-inflammatory combination therapy, it might provide an attractive approach to further increase efficiency in preventing disease progression in hypertension-induced cardiovascular damage.

## Data Availability

Not applicable.
